# Functional adaptation after femoral intertrochanteric valgus osteotomy in Legg–Calvé–Perthes disease

**DOI:** 10.1038/s41598-023-45749-1

**Published:** 2023-11-23

**Authors:** Ferdinand Wagner, Barbara Weiß, Boris Michael Holzapfel, Christian Max Ziegler, Bernhard Heimkes

**Affiliations:** 1 Department of Orthopedics and Trauma Surgery, Musculoskeletal University Center Munich, University Hospital, Ludwig-Maximilians-Universität, Munich, Germany; 2 Department of Pediatric Surgery, Dr. Von Hauner Children’s Hospital, Ludwig-Maximilians-Universität, München, Munich, Germany; 3 Institute of Health and Biomedical Innovation, Queensland University of Technology (QUT), Brisbane, Australia; 4 Orthopaedic Department, Klinikum Stuttgart Olgahospital, Kriegsbergstrasse 62, 70174 Stuttgart, Germany; 5 Department of Pediatric Surgery, Pediatric Orthopedic Section, Klinikum Dritter Orden, Menzinger Straße 44, 80639 Munich, Germany

**Keywords:** Paediatric research, Medical research, Outcomes research, Skeleton

## Abstract

Legg–Calvé–Perthes disease (LCPD) requires individualized treatment in order to regain a functional hip joint. In severe cases, in which a congruent joint cannot be achieved, other options are necessary in order to improve functionality and prevent early osteoarthritis. Therefore, we analysed the clinical and radiologic outcome of 28 patients after valgus osteotomy of the proximal femur (VOF). We examined the range of hip motion, functionality and health-related quality of life (HRQoL) via modified Harris Hip Score (mHHS) and Kidscreen-10. Radiographic analysis contained quantitative and qualitative measurements of hip morphology. In particular, we correlated the results with the change of the pelvic-femoral angle (PFA). PFA was defined as the angle between the anatomical diaphyseal line of the femur and a vertical line through the pelvis. The mean follow-up was 5.5 years. Patients showed high mHHS and good HRQoL postoperatively. An increase in ROM with an improvement of 30.5° abduction and 10.3° internal rotation was evident. PFA correlated with adduction contracture and improved significantly after surgery. In consideration of careful patient selection, VOF showed a positive effect on ROM, pain, HRQoL, radiographic congruence and outcome. We identified the age at surgery and an increasing adduction contracture—objectified by a decreased PFA—as a prognostic factor.

## Introduction

**Figure 1:**
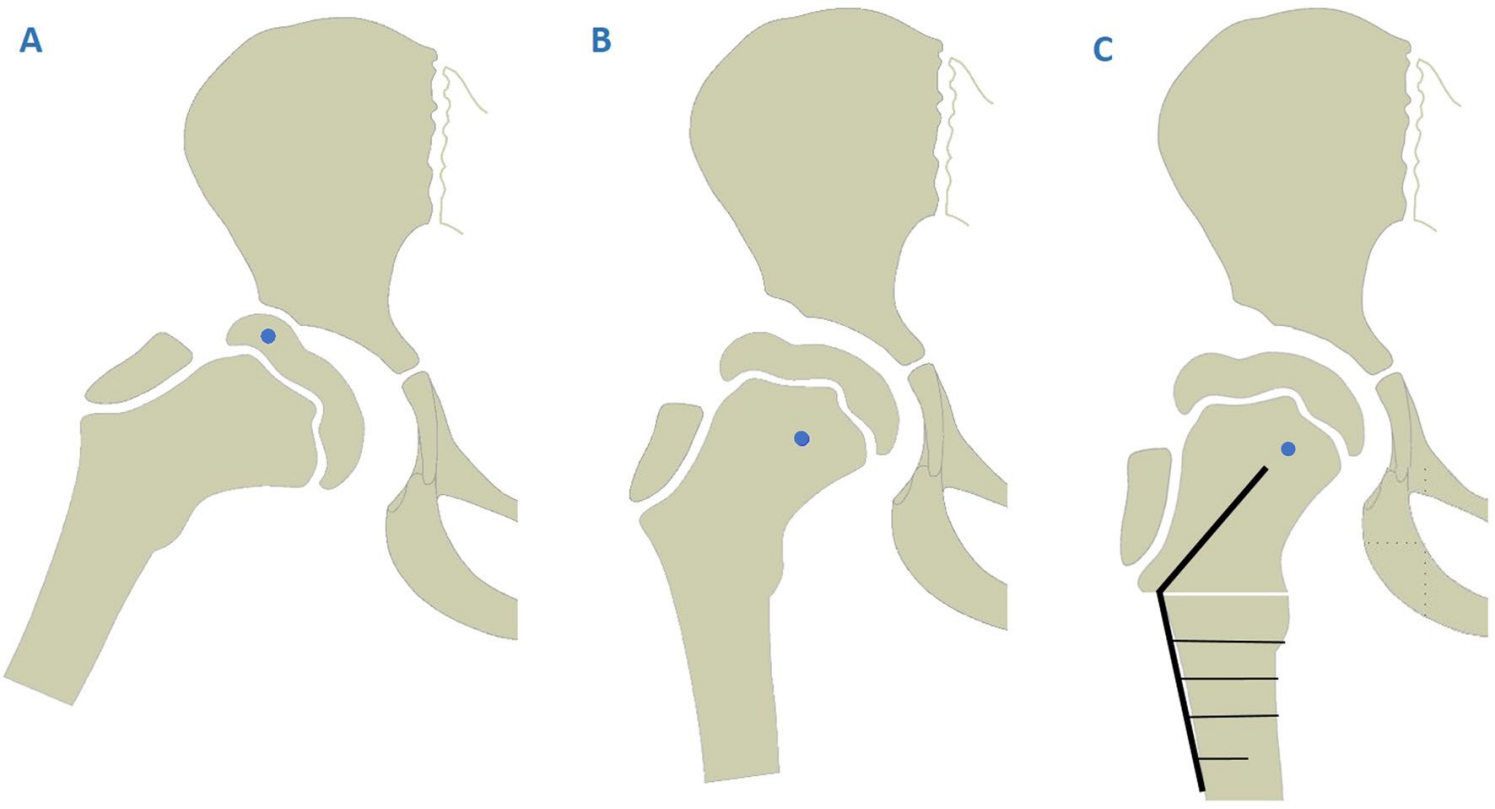
Deformed and enlarged femoral head with hinge abduction phenomenon. (**A**) In abduction the lateral part of the femoral head hinges against the acetabular rim; filled circle = lateralized centre of rotation. (**B**) Adduction of the hip demonstrates the conditions with VOF: filled circle = centered centre of rotation. (**C**) Proceeded proximal femoral valgus osteotomy resulting in a distancing of the lateral femoral deformation and therefore reducing hinge abduction in a normal standing position.

Therapeutic options for Legg–Calvé–Perthes disease (LCPD) are in continuous discussion. International consensus is the principle of containment therapy with the aim of a congruent hip joint and a centred femoral head in order to decrease the risk of the hinge abduction phenomenon (HA) and to maintain a satisfying hip function. Pathologic healing into an incongruent hip joint mostly leads to pain, limited range of motion (ROM) and early osteoarthritis. HA is a sign for poor prognosis due to incongruence of the hip joint caused by an enlarged extruding head, which impinges at the lateral rim of the acetabulum. Anthony Catterall was the first to describe HA to be associated with severe cases of LCPD in 1981^1^. In his late period Catterall proposed valgus osteotomy of the femur (VOF) as a salvage procedure^2,3^. VOF readjusts the enlarged and protruded femoral head within the acetabular centre, prevents the continuous and repetitive strike against the acetabular rim and rotates the remaining solid and mechanical stable areas of the epiphysis into the load bearing sectors (Fig. 1). This aims to reduce pain, to regain abduction and to postpone further collapse of the epiphysis as well as the need for endoprosthetic total hip arthroplasty in the usually young patients^1–3^.

The purpose of this retrospective study was to evaluate the indication and the results of VOF in our patients with LCPD considering clinical and radiographic variables. We defined the pelvic-femoral angle (PFA) and analysed its clinical value for the treatment in LCPD.

## Patients and methods

Data of 28 patients who underwent VOF for LPCD in our university hospital between January 2004 and June 2013 were analysed retrospectively including clinical records and radiographs. Indication for VOF was given for non-containable hips with pain, extrusion of the overgrowing femoral head, progressive restriction in hip abduction or eventual hinge abduction. Arthrographic evidence of an improved containment in hip adduction confirmed the indication.

### Ethics

Written informed consent was obtained from all subjects and/or their legal guardians to participate in the study. The data analysis was approved by the local ethics committee of the Ludwig-Maximilians University (Ethics No: 95-14) and conducted according to the guidelines of the Declaration of Helsinki (Votum No. 95-14, 05.06.2014). Written consent to the operation of all parents or legal representatives of the patients were available.

### Clinical evaluation

The modified Harris Hip Score (mHHS)^4^ and the Kidscreen-10^5^ were utilized at the latest follow-up for standardized evaluation of the health related quality of life (HRQoL), physical and psychological well-being as well as the subjective functional outcome. Pre- and postoperative ROM was assessed and compared. Measurements were performed preoperatively (preOP), postoperatively (postop) and while latest follow-up (LFU) in order to evaluate the clinical and radiological course of the disease under therapy.

### Radiographic assessment

Utilizing approved and standardized methods of measurement^6,7^ radiographic assessment included measurements evaluating the femoral head and neck deformity such as neck-shaft angle (NSA), articulo-trochanteric distance (ATD)^8^, sphericity index of Mose (SI)^9^, deformity index of Nelson (DI)^10^, Stulberg classification^11^, acetabular angle of sharp (AAS) and signs of femoral head extrusion such as acetabulum-head quotient (AHQ)^12^, epiphyseal extrusion index (EEI)^13^ and tear drop distance (TDD)^14^. Remodelling of the femoral head was defined by the change of DIN preOP to LFU according to Yoo et al.^15^.

**Figure 2:**
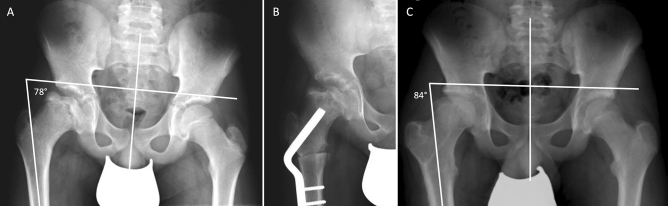
(**A**) Definition of the pelvic-femoral angle (PFA): angle between the anatomical diaphyseal line of the femur and a vertical line through the pelvis in the a.p. view (from the left to the right anterior inferior iliacal spine). The image shows a 11-year old patient. (**B**) Postoperative X-ray during VOF of the right hip 6 weeks after surgery. (**C**) Final X-ray after full maturation of the patient (17 years). The initial PFA of 78° was increased to 84°.

In order to determine hip function radiologically, we introduced the pelvic-femoral angle (PFA) as a measure for hip adduction/abduction. This new parameter was defined as the angle between the anatomical diaphyseal line of the femur and a vertical line through connecting the left and right anterior inferior iliacal spines of the pelvis in the a.p. view (Fig. 2). Therefore, x-rays were acquired placing the patients in supine position and the affected extremity was brought into maximum abduction. Legs were internally rotated by 15 degrees in 0 degrees hip extension.

### Statistical analysis

Statistical analysis was performed with SPSS v23. Student’s t-test was used to analyse changes in preoperative and postoperative variables and differences between groups. Correlation between variables was verified with Pearson or Spearman or Kendalls Tau test, depending on the scale. Kidscreen-10 questionnaire was evaluated with the Rasch model using person parameter estimates.

## Results

Twenty eight patients met the study criteria and were assessed using the above-mentioned methods. 23 boys and 5 girls with a mean age of 10.7 years (range 6.2–16.3 years) at time of surgery were followed-up for an average of 5.5 years (= latest follow-up = LFU; range 1.0–10.5 years). 9 patients underwent VOF with simultaneous Salter’s pelvic osteotomy.

Seven patients were classified as “residual stage” at the time point of surgery. 21 patients underwent VOF before residual stage and were therefore classified according to Catterall and Herring (see Table 1 for details)^7,16^. Ten patients underwent previous surgical treatment such as pelvic osteotomy (n = 2), varus osteotomy of the proximal femur (n = 6), adductor tenotomy (n = 1) and combined pelvic osteotomy with varus osteotomy of the proximal femoral (n = 1).

**Table 1:** Patient’s characteristics.

Patient’s characteristics		
Number of patients	28	
Male:female	23:5	
Mean age at onset of LCPD symptoms [years]	8.2 (3.2–14.9)	
Mean age at surgery [years]	10.7 (6.2–16.3)	

	N	[%]
Staging at diagnosis		
Initial	3	10.7
Fragmentation	14	50.0
Re-ossification	4	14.3
Residual stage	7	25.0
Catterall classification		
I	0	0
II	5	17.9
III	11	39.3
IV	5	17.9
Herring classification		
A	0	0
B	7	25.0
C	14	50.0
Stulberg classification at LFU		
I	1	3.8
II	3	11.5
III	12	46.2
IV	8	30.7
V	2	7.7

### Questionnaires

The response rate to questionnaires was 53.6%. Mean modified Harris Hip Score (mHHS) revealed 92 out of 100 points examining pain and hip function postoperatively (Table 2). The category “function/activity of daily life” also showed excellent results at LFU (max. 47 points).

**Table 2:** Results of the mHHS of the operated hip.

	Points	N = 15
Total mHHS of the operated hip		
Excellent	90–100	11
Good	80–89	2
Average	70–79	0
Poor	< 70	2
Mean	92	
Pain		
None	44	12
Slight pain	40	1
Mild pain	30	1
Moderate pain	20	0
Marked pain	10	1
Totally disabled	0	0
Mean	40.5	
Function/activity of daily life		
	33	1
	36	2
	40	1
	41	2
	44	4
	47	5
Mean	42.5	

**Figure 3:**
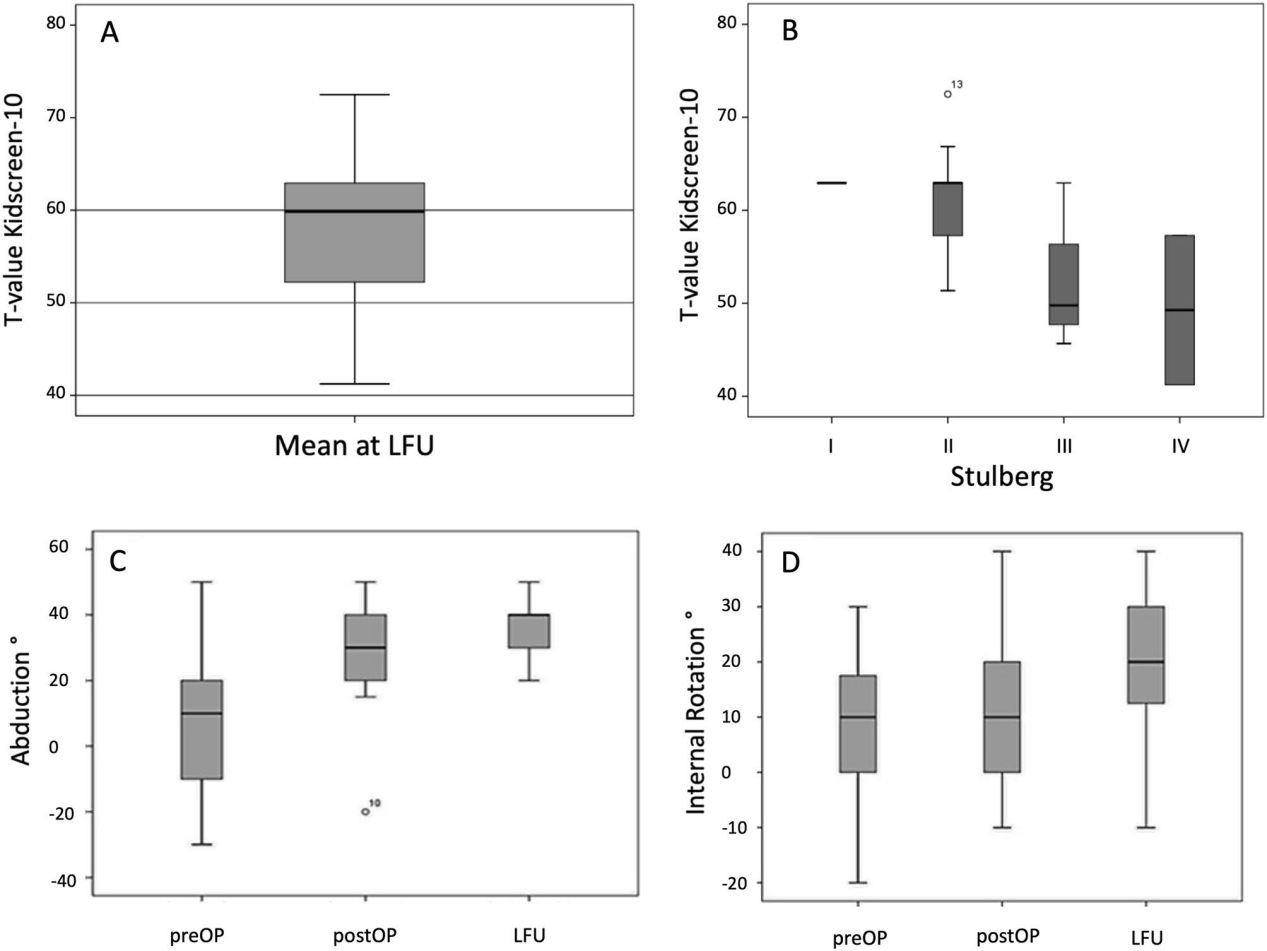
(**A**) T-values of Kidscreen-10 at LFU and (**B**) T-values grouped by the Stulberg classification. Change in clinically evaluated abduction (**C**) and internal rotation (**D**) preOP, postOP and at LFU. Boxpolts are defined as bottom and top of box = 25th and 75th percentiles, thick line inside box = mean, thin line inside box = median, I bar = maximum and minimum of data, and circle with slash = outlier.

The Kidscreen-10 revealed high HRQoL of patients with a mean T-value of 58.0 (StDev ± 8.4; min 41.2; max. 72.5) at LFU (Fig. 3). Statistical analysis showed significant correlation of Kidscreen-10 with Stulberg classification revealing that patients with lower Stulberg stages have a better Kidscreen-10 score (r =  − 0.559; p = 0.030; Fig. 3).

### Clinical examination

Mean time point of acquisition of ROM was 5.5 years after surgery (LFU). There was a significant increase in ROM in the initial postoperative phase (postOP). 10 patients showed an adduction contracture preoperatively (preOP). Particularly abduction and internal rotation of the hip showed significant improvement with an increase of + 30.5° abduction (p ≤ 0.000, Fig. 3) and + 10.3° internal rotation (min − 10.0°; max + 60.0°; p = 0.032). Regarding internal rotation 4 patients showed a decrease postOP compared to preOP. However, the postoperative course of abduction remained stable without a significant change until LFU (p = 0.193). Significant correlation of a restriction in ROM and PFA (p < 0.01) was found. No significant difference in mean leg length from postOP to LFU could be detected clinically (p = 0.135).

### Radiologic analysis

#### Neck-shaft-angle and limb length discrepancy

The preoperative neck-shaft angles of the operated side were in the standard age-related range of the general population (50) (see Table 3). Postoperatively, a significant valgus position of the operated hip joint was achieved (p = 0.003). NSA at LFU did not differ significantly from postOP indicating no loss of correction over time.

**Table 3:** (A) Results limb length discrepancy (LLD; affected side) and (B) NSA preOP, postOP and at LFU of the affected limb.

A	LLD preOP N = 24		LLD at LFU N = 22	
Mean [cm] (± StDev)	− 1.0 (± 1.4) Min − 5.0; Max + 1.0		0.6 (± 1.2) Min − 3.0; Max + 1.5	

B	NSA preOP (N = 28)	Planned valgisation (n = 26)	NSA postOP (N = 28)	NSA at LFU (N = 28)
Mean[°]	129.5 (± 10.0)	28.2 (± 8.8)	152.5 (± 2.1)	149.0 (± 10.4)

C	Correlation with LLD (r)	Significance (p)		
Radiologic valgisation angle	0.554	0.007*		
NSA at LFU	0.444	0.038*		

(C) Correlation of LLD with the intraoperatively implemented valgisation angle and postoperative NSA.

*Indicating significance p < 0.05.

The pre- or intraoperatively calculated valgisation angle and the actual surgically applied osteotomy angle (mean 28°) differed by 5° in average (p = 0.011).

#### Pelvic-femoral angle (PFA)

**Figure 4:**
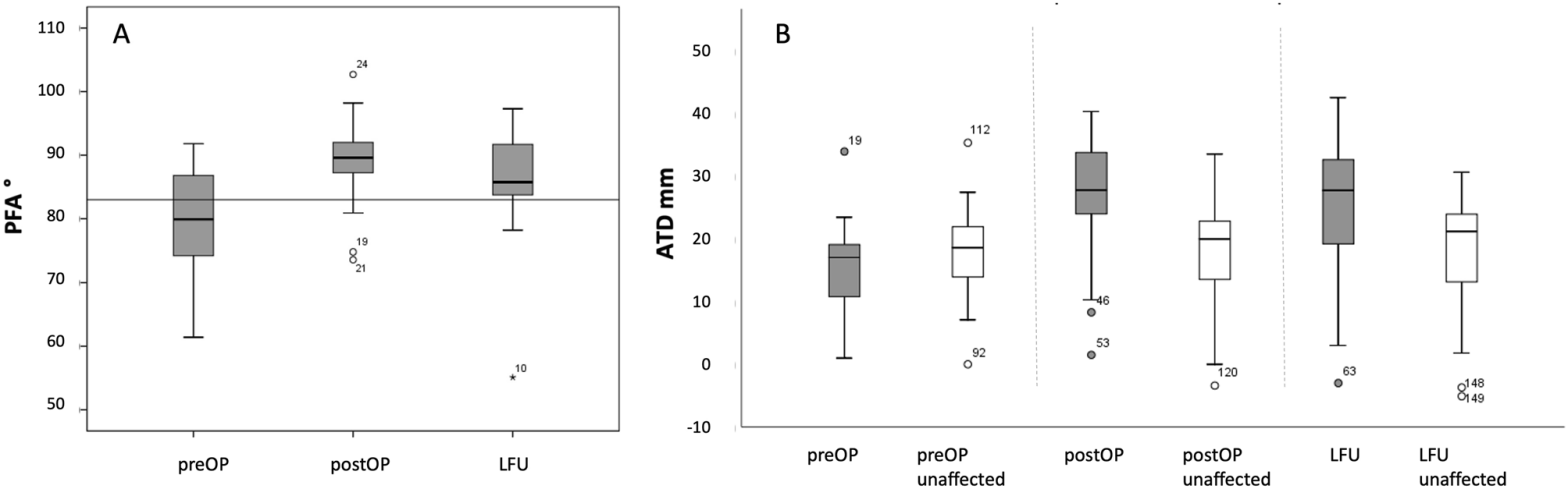
(**A**) PFA over time preOP, postOP and at LFU. (**B**) ATD over time preOP, postOP and at LFU compared to the unaffected side. For significant differences see Tables 4 and 5. Boxpolts are defined as bottom and top of box = 25th and 75th percentiles, thick line inside box = mean, I bar = maximum and minimum of data, and circle with slash = outlier.

The change in PFA between preOP and postOP as well as preOP and LFU was both statistically significant at each side (p ≤ 0.001; Fig. 4A, Table 4). There was a significant difference when comparing the operated and contralateral side preOP, but not if comparing both sides at LFU (p = 0.473) suggesting a long-term normalization of PFA due to VOF (Additionally, we determined a significant correlation of preOP PFA with clinical preOP abduction when utilizing Pearson Correlation test. Same was found when calculating the correlation of the difference of preOP and postOP PFA vs. the increase in abduction due to surgery.

**Table 4:** (A) Pelvic-femoral angle (PFA) preOP, postop and at LFU.

A	Operated side	Contralateral side	Operated side	Contralateral Side	Operated side	Contralateral side
Pelvic-femoral angle (PFA)	preOP		postOP		LFU	
Mean (°)	79.7 (± 8.0)	90.0* (± 8.9)	89.1** (± 6.6)	82.7*** (± 6.0)	86.7****(± 8.1)	85.2 (± 7.8)
Minimum (°)	61.4	73.5	73.6	68.1	55.1	68.4
Maximum (°)	91.8	125.5	102.7	99.1	97.3	114.5

n = 28		*p < 0.001 vs operated preOP	**p < 0.001 vs operated preOP	***p < 0.001 vs operated postOP	****p = 0.002 vs operated preOP	p = 0.473 vs operated at LFU
B			Pearson correlation (r)		Significance (p)	
PFA preOP vs. abduction preOP			0.781		≤ 0.001*	
Difference PFA Pre/postOp vs. Increase Abduction			0.637		≤ 0.001*	

(B) Pearson Correlation of PFA and abduction.

Asterisks (*) indicate statistical difference (see Table text).

#### Articulo-trochanteric-distance

Mean values for articulo-trochanteric distance (ATD) were in the normal range and without significant change comparing preOP, postop and at LFU on the unaffected side. However, a significant increase in mean ATD was found at the operated side over time (p < 0.001; Fig. 4B). Postoperatively, there was also a significant difference when comparing the operated and the unaffected side at the time points postOP and LFU (see Table 5 for p-values).

**Table 5:** ATD of affected side and correlating p-values for ATDs.

n = 28	ATD unaffected [mm]			ATD VOF [mm]		
	preOP	postOP	LFU	preOP	postOP	LFU
Mean	17.6 (± 7.4)	17.7 (± 9.2)	17.9 (± 9.6)	15.4 (± 6.7)	26.9 (± 9.2)	25.4 (± 10.8)

ATD at the operated side (VOF)		Significance (p)				
ATD preOP	ATD postOP	< 0.001*				
ATD preOP	ATD LFU	< 0.001*				
ATD postOP	ATD LFU	0.548				
ATD preOP VOF	ATD preOP contralateral	0.254				
ATD postOP VOF	ATD postOP contralateral	< 0.001*				
ATD LFU VOF	ATD LFU contralateral	0.009*				

*Statistical difference (p ≤ 0.001).

#### Deformity index of Nelson (DIN)

We found a significant negative correlation of DIN preOP with the Herring stage (r =  − 0.558; p = 0.013). Preoperatively, 5 patients had a DIN < 0.3 (= regarded as normal), postoperatively 6 and at LFU 10 patients. “Remodelling” was defined as a significant difference between DIN preOP and DIN at LFU. Analysis showed no difference regarding remodelling if correlated with the Stulberg groups. The lack of a significant difference of DIN might be due to a lack of number of cases per Stuhlberg group. Nevertheless, remodelling correlated negatively with age at surgery and with Herring staging (Table 6).

**Table 6:** (A) Patients with a normal or mild DIN (< 0.3) at LFU.

A				
Patient ID	Stulberg Group	DIN preOP	DIN LFU	Remodelling
4	II	0.33	0.18	− 0.15
9	II	0.28	0.27	− 0.01
17	III	0.31	0.14	− 0.17
19	V	0.51	0.12	− 0.39
34	III	0.43	0.27	− 0.16
38	III	0.14	0.27	+ 0.13
43	III	0.58	0.14	− 0.44
47	IV	0.24	0.28	+ 0.04
49	III	0.06	0.2	+ 0.14
74	III	0.52	0.17	− 0.35

B		Correlation (r)	Significance (p)	
DIN preOP vs. herring stage		− 0.558	0.013*	
Remodelling vs. age at surgery		− 0.448	0.025*	
Remodelling vs. herring stage		− 0.412	0.041*	

(B) Correlation of remodelling. Remodelling = DIN at LFU–DIN preOP.

### Additional radiologic parameters

We also performed a thorough analysis in changes regarding preOP and postOP acetabulum head index, tear drop distance, acetabular angle of sharp. No significant changes were found for these parameters preOP and postOP (data not shown). However, we found clear radiological signs of extrusion: 61% had preoperatively pathological tear drop distance values and 81% had a poor prognosis as determined by an increased epiphyseal extrusion index.

## Discussion

In severe courses of LCPD therapy options are limited. The postulated aim of hip containment mostly requests invasive methods because of an enlarged and extruded femoral head. In his late active period Catterall proposed VOF as an appropriate salvage procedure with satisfactory results^2,3^. Since VOF is not a standard method in the treatment of LCPD, only limited comparative studies are available. Nevertheless, the indication for VOF already in an early stage of the disease was described by few authors^2,3,15,17,18^. A special point of interest however is the benefit of early VFO on containment, pain and hip function, along with associated variables. Therefore, this study examined the outcome of VOF in severe cases of LCPD at the residual stage as well as early active stages in connection with PFA.

The mHHS revealed good hip function after VOF and the best result eleven times with an average of 92 out of a maximum of 100 points. It showed similar results compared to Palmen et al., Bankes and Yoo et al. who used the Iowa Hip Score to assess pain and hip function^2,15,17,19^.

Improvement or at least maintenance of ROM is one of the most important goals of treatment. Our study showed an excellent improvement of hip abduction and internal rotation comparing the preOP and postOP clinical status. Previous studies confirmed these results. Yoo et al. significantly increased hip abduction and internal rotation via VOF until LFU (p ≤ 0.002)^15,17^. Bankes et al. showed significant reduction of fixed adduction 1 year after surgery (p < 0.0001)^2^.

We used Kidscreen-10 as a national and international validated, standardized instrument for health-related quality of life^5^ and revealed a high HRQoL with a mean T-value of 58.0. This indicates that the children feel happy, fit and content in relation to family life, peer groups and everyday school life. Palmen et al. also showed a higher HRQoL in children with LCPD compared to the healthy comparison group^19^. The clear negative correlation with the Stulberg result speaks for the connection between the radiological and clinical results. A poorer Stulberg stage, and thus a femoral head with a more pronounced deformity, influences the patient’s HRQoL life more than a lesser deformity.

We found no pathological values regarding ATD on the non-operated side, which confirms a healthy side suitable as controls. The operated side showed significantly higher mean values in the postOP measurement than preOP. The change in the difference between the pre- and postOP mean values correlated significantly with the radiologically measured increased NSA after surgery. The measurements thus confirm the increase in the distance between the femoral head and the greater trochanter, which is expected as a result of the valgisation of the femoral neck. Of note, pathological values of the ATD, caused by the still active disease process and the subsequent hip deformity do not necessarily appear in the preoperative measurement. Edgren et al. also found that reduced ATD values only occurred in the repair stage, when the femoral head already had reached its final shape^20^. In terms of biomechanical forces, VOF corresponds to a trochanter distalization. This improves the preoperative unfavourable lever and pressure conditions in the hip joint and increases the muscular pre-tension postoperatively^21–23^.

According to Quain and Catterall, the valgus osteotomy of the femur can be expected to have a beneficial effect on the difference in leg length^1^. Our results regarding LLD over time are not statistically significant. However, an additional shortening osteotomy of the proximal femur was performed in 8 patients in order to compensate for a preoperatively difference in leg length. These patients showed a smaller LLD (pre- and postoperative), but in average a larger valgus angle (although not statistically significant). Banke's investigations were able to achieve a postoperative reduction in LLD, but equally without statistical significance (p = 0.12)^2^. A correlation of LLD with a restriction of hip joint mobility or the severity of the disease also could not be demonstrated in our study. Nevertheless, our results speak for the fact that this matter has to be addressed carefully in pre- and intraoperative planning.

This study demonstrates that a significant normalization of the femoral neck function can be achieved via VOF when comparing the NSAs of the operated side before and after the operation. The NSAs at the measured time points, the planned correction angle and the postoperative change in NSA were comparable with the results of Bankes et al. and Sidler-Maier et al.^2,24^. No correlation was found between the age at the time of surgery and the postoperative change in the corrected NSA until LFU (p = 0.656). Therefore, we assume that the postOP result is maintained, even taking into account the continuing growth trend in the number of patients under the age of 18^25^. Most likely, one has not to consider a significant overcorrecition when planning the valgisation angle.

We could not find a statistically significant correlation of DIN with the Stulberg result. It must be noted, however, that after we separated the groups into Stulberg I and II and Stulberg III and IV—as proposed by Nelson—there were only four patients in the first group for evaluation^10^. Nevertheless, there was a significant, negative correlation with the Herring stage. This indicates, that a more severe pathological femoral head involvement is associated with a more “de-rounded” femoral head. At the time of surgery 17 patients were in the condensation or fragmentation stage according to Waldenström. For these stages of the disease, a higher remodelling potential of the femoral head is assumed, which is present until the early stage of repair^26^. Yoo et al. treated about twice as many Catterall Stage IV patients than us (61.3% vs. own 23.8% vs.) and found significant remodelling^3,15^. The authors therefore expanded the indication for valgus osteotomy of the femur. Instead of just treating healed, off-centre and incongruent hip joints, the operation was also performed in all stages of the disease if an irreversible hinge-abduction phenomenon occurred and if there was improvement in congruence in the hip adduction position. Yoo et al. defined the change in DIN from preoperative to the last follow-up time point as the “remodelling” of the femoral head^15^. In our own investigations, a correlation was found between remodelling and the age at the time of surgery and the Herring stage. This is in line with Yoo et al. who also found a correlation between the remodelling and the age at the time of the operation. The equally significant correlation with staging speaks for a higher potential of remodelling in younger patients and the earlier the surgical intervention took place in the course of the disease. Of note, DIN does not assess the degree of pathological congruence^19^.

In this study, we implemented a radiologic measure to quantify restriction of ROM. We demonstrated a statistically significant connection between ROM restriction and PFA. Furthermore, a correlating improvement of ROM as well as PFA could be achieved through the surgical treatment. Seven patients showed a reduced PFA and an adduction contracture preOP which was successfully compensated by VOF. Additionally, the highest number of normal PFA measurements in the affected hips was found at LFU compared to preOP values. The adduction position of the diseased hip in the X-ray images as a preliminary stage of the HA phenomenon could be clinically proven. The newly introduced PFA can serve as a quantifiable radiological measurement value for adduction contracture. However, one must strictly pay attention that the diseased leg is positioned in maximal possible abduction as this might significantly influence the quality of the measurement.

The adduction contracture can be seen as an early clinical head-at risk-sign of LCPD^3,27^. ROM is not only important for the physiological wellbeing of the patients. It is also a prognostic factor and can be an early sign for hip deformity and extrusion of the femoral head. The insufficiency of the hip abductors due to an elevated position of the greater trochanter can be seen as equivalent to a hip adduction contracture and is of special interest in these patients^28^. In conclusion, we found a positive effect on remodelling of the femoral head in our patients due to VOF and therefore due to an improvement of ROM clinically and PFA radiologically. This has to be considered regarding the indication for invasive procedures.

We therefore postulate an expansion of the indication for VOF not only in the presence of an already present hinge abduction phenomenon. We do not see the surgical method as a sole “salvage procedure” but as a way of improving containment and thereby improving clinical symptoms, hip joint mobility, the biomechanical conditions at the hip joint and quality of life. This achieves the essential therapy goals of LCPD^2,3,26^. PFA is a new parameter to help in decision making for the often uncontrollable course of the disease and has to be evaluated further in larger study cohorts.

## Data Availability

The data stays with the authors due to ongoing studies but will be shared upon any individual request. For requests, the corresponding author (B.H.) can be contacted at any time.
